# Factors associated with treatment compliance among middle-aged patients with type 2 diabetes mellitus in King Abdulaziz University Hospital, Jeddah, Saudi Arabia

**DOI:** 10.3389/fcdhc.2026.1824629

**Published:** 2026-06-17

**Authors:** Abdulrahman Abudawood, Leen Algaidy, Manal Almazroui, Shaima Albeladi, Arwa Albeladi, Lama Alnamani, Bedoor Alghanmi, Iman Wahby, Salhah Alsulami

**Affiliations:** 1 Faculty of Medicine in Rabigh, King Abdulaziz University, Rabigh, Saudi Arabia; 2 Faculty of Medicine in Rabigh, King Abdulaziz University, Jeddah, Saudi Arabia; 3 Department of Community Medicine, Faculty of Medicine, King Abdulaziz University, Jeddah, Saudi Arabia; 4 Department of Medicine, Faculty of Medicine in Rabigh, King Abdulaziz University, Jeddah, Saudi Arabia

**Keywords:** comorbidities, medication adherence, middle-aged patients, Saudi Arabia, type 2 diabetes mellitus

## Abstract

**Background:**

Type 2 diabetes mellitus (T2DM) is a chronic condition requiring sustained medication adherence and lifestyle modification. In Saudi Arabia, T2DM affects 21–24% of the population, with prevalence projected to rise by 2030. Demographic factors and comorbidities pose significant challenges to treatment compliance, particularly among middle-aged adults.

**Objective:**

To examine factors influencing treatment adherence among middle-aged adults (40–65 years) with T2DM at King Abdulaziz University Hospital, Jeddah.

**Methods:**

An analytical cross-sectional study was conducted at King Abdulaziz University Hospital, Jeddah. Data were collected via face-to-face interviews using a validated bilingual questionnaire covering demographics and treatment compliance. A total of 393 participants were enrolled, exceeding the minimum calculated sample of 365 (95% confidence level, 5% margin of error). Data were analysed using SPSS Version 20, with statistical significance set at p < 0.05.

**Results:**

Suboptimal adherence was reported by 78.4% of participants. Univariate logistic regression identified five predictors of suboptimal adherence: absence of treatment plan clarification (OR 3.197), lack of explanation of diabetes complications (OR 3.120), discontinuation of medication without consulting a provider (OR 0.130), non-commitment to medication dispensing dates (OR 0.139), and non-commitment to doctor appointments (OR 0.093). Multivariate backward stepwise logistic regression identified four independent predictors: age 51–55 years was associated with reduced odds of suboptimal adherence compared to age 40–45 years (aOR 0.235; p = 0.027); second-hand smoke exposure was associated with reduced odds compared to non-smokers (aOR 0.295; p = 0.027); working more than 55 hours per week was associated with reduced odds compared to working fewer than 35 hours (aOR 0.141; p = 0.038); and a diabetes diagnosis duration of 6–10 years was associated with increased odds compared to 1–5 years (aOR 2.553; p = 0.047).

**Conclusion:**

Adherence to anti-diabetic therapy remains suboptimal among middle-aged Saudi adults. Key contributors to poor adherence include missed appointments, unclear treatment plans, inadequate education on diabetes complications, and self-discontinuation of medications.

## Introduction

Type 2 diabetes mellitus (T2DM) is a metabolic disorder characterised by impaired insulin secretion or sensitivity arising from a combination of genetic predisposition and environmental factors such as excessive food intake, physical inactivity, obesity, stress, and ageing (1). T2DM is a chronic disease requiring complex pharmacological therapy to control hyperglycaemia, alongside lifestyle and dietary modification (2).

However, patients often encounter difficulties because T2DM produces few subjective symptoms, making it challenging to sustain motivation for long-term treatment. As a result, many patients miss their medical appointments or struggle to adhere to their medication regimen (3, 4). Treatment compliance has been defined in the literature as “the level or extent of adherence to the provider’s daily treatment instructions with regard to schedule, dosage, and frequency” (5).

Demographic factors, such as age and sex, were identified as being associated with medication adherence (6). Patients of all ages are concerned about medication adherence, but older adults are more likely than younger adults to experience pharmaceutical non-adherence because of various chronic diseases, polypharmacy, and cognitive impairment (7).

Furthermore, comorbid conditions such as hypertension, cardiovascular disease, and chronic kidney disease commonly emerge over the course of type 2 diabetes mellitus (T2DM). These comorbidities may limit patients’ capacity to attend routine clinical appointments and are associated with reduced adherence to prescribed therapeutic regimens. Prior studies have consistently reported a significant relationship between the presence of comorbidities and suboptimal medication adherence among individuals with T2DM (8).

The Kingdom of Saudi Arabia (KSA) has one of the highest rates of diabetes mellitus in the Middle East; according to a previous meta-analysis, the prevalence of T2DM in KSA is estimated at 28% (9). KSA ranks seventh worldwide and second in the Middle East. Approximately seven million individuals in the country have diabetes, and three million are pre-diabetic (10). If conditions remain unchanged, the number of adults with DM is expected to rise by 2030, making up one-fourth of the population (11).

In the Asir region, the southern region of KSA, a population-based study investigating complication awareness and its relationship to medication compliance was conducted. The results showed that good awareness levels were found in 54.1% of patients aged 25–35 years compared with 35.1% of those older than 50 years. In addition, 62.5% of patients diagnosed for less than one year were well aware of the potential consequences of diabetes, compared with 51.3% of those who had it for more than ten years (12). In the Western region of Saudi Arabia, medication adherence has been evaluated in primary healthcare centres and was found to be suboptimal (13).

As observed in other settings, age and educational attainment play an important role in medication adherence. A key knowledge gap remains, however, as medication adherence among middle-aged patients with diabetes in tertiary care hospitals in the Western region of Saudi Arabia has not been previously assessed. Therefore, this study aimed to investigate factors associated with treatment compliance among middle-aged adults with type 2 diabetes.

## Materials and methods

This study was an analytical cross-sectional study conducted at King Abdulaziz University Hospital (Outpatient Clinics) in Jeddah, Saudi Arabia. The study targeted a specific population, including male and female patients diagnosed with diabetes aged 40–65 years attending the outpatient clinics. Patients outside this age range were excluded. A consecutive sampling strategy was employed: all eligible patients attending the outpatient diabetic clinics during the data collection period who met the inclusion criteria were approached sequentially and invited to participate in face-to-face interviews until the required sample size was reached. This approach was chosen because it minimises selection bias within the clinic setting and reflects the actual patient flow during the study period. Data were collected using a structured, validated, and widely used questionnaire across the Arab world, Iraq anti-diabetic adherence scale (IADMAS). The IADMAS consists of eight items, each scored dichotomously (0 = non-adherent behaviour; 1 = adherent behaviour), yielding a total score ranging from 0 to 8, where higher scores reflect greater adherence. IADMAS is a valid and reliable tool to assess anti-diabetic medication adherence among Iraqi patients with Cronbach’s alpha of 0.712 (13). Although the IADMAS was originally developed and validated in an Iraqi Arab population, it has subsequently been used in Saudi Arabian studies of medication adherence in T2DM patients, demonstrating acceptable reliability in this culturally and linguistically comparable setting (13). The questionnaire was administered in its validated Arabic version, which is the native language of the study population, further supporting its appropriateness for use here.

The total IADMAS score ranged from 0 to 8, with medication adherence categorized into three levels: high adherence (score = 8), moderate adherence (score 6 to <8), and low adherence (score <6) (13). For the purpose of analysis participants were categorized into two groups: optimal adherence (score = 8; all eight items answered in the fully adherent direction) and suboptimal adherence (score <8; combining moderate adherence [score 6 to <8] and low adherence [score <6]). This binary cut-off was adopted to facilitate logistic regression analysis and to maintain consistency with prior studies applying the IADMAS in comparable clinical settings (13).

The questionnaire was available in both Arabic and English, and was divided into two main sections. The first section gathered demographic information, such as age, gender, marital status, education level, employment, income, and smoking habits. The second section focused on treatment compliance, covering factors such as medication adherence, lifestyle behaviours, and financial barriers.

Prior to participation, informed consent was obtained. The research team introduced themselves and explained the study’s purpose, including the questionnaire’s topic, expected duration of approximately 15 minutes, and the voluntary nature of participation. Participants were informed that they could withdraw at any time without consequences. In addition, no direct benefit will be attained upon enrolment. Access to principal investigators was given upon requests. Ethical approval for the study was obtained on 19/02/2024 from King Abdulaziz University Bioethics Unit (Reference No. 27-24), and participant recruitment was done using consecutive sampling technique.

The minimum sample size for the study was calculated using OpenEpi, Version 3, with a 95% confidence level and a 5% margin of error, yielding a required minimum of 365 participants. The following formula was used to calculate the sample size:


n = [DEFF×N×p(1−p)]/[(d2/Z2  1−α/2×(N-1))+p×(1−p)]

$$\begin{array}{c} \text{N }=\;20000,\text{ hypothesized }\%\text{ frequency of outcome factors}\\ \text{ in the population }(\text{p})\;=\;41.5\%\;+\;/-5. \end{array}$$

Confidence limits as % 100 (absolute +/− %)

D: the maximum acceptable error=5%

Design effect (for cluster surveys−DEFF):1

A 10% pilot study was conducted to test the methodology but was excluded from the results. The minimum sample size needed was calculated to be 365. To account for potential non-response and incomplete questionnaires, additional participants were recruited, resulting in a final analysed sample of 393 participants.

Data management and analysis followed a structured process. Data analysis was performed using SPSS Version 20. Descriptive statistics, including frequencies and percentages, were used to summarize demographic data, while the Chi-square test assessed associations between various factors and treatment compliance. Statistical significance was set at a p-value of <0.05. Univariate binary logistic regression was first performed to calculate crude odds ratios (ORs) and 95% confidence intervals (CIs) for each variable. Variables that were statistically significant in univariate analysis (p < 0.05) were subsequently entered into a multivariate binary logistic regression model using a backward stepwise approach to identify independent predictors of suboptimal adherence, yielding adjusted odds ratios (aORs). ORs greater than 1 indicate increased odds of suboptimal adherence, whereas ORs less than 1 indicate a protective association (i.e., reduced odds of suboptimal adherence).

## Results

**Table 1** shows the sociodemographic and clinical characteristics of the 393 participants. The sample had a mean age of 53 years and was predominantly female (66.7%) and married (69%), with approximately half in employment. Nearly half (46.1%) had at least one comorbid condition, hypertension being the most prevalent, and 44.5% were taking additional medications alongside their antidiabetic regimen. Detailed frequency distributions for all variables are presented in **Table 1**.

**Table 1:** Sociodemographic and clinical characteristics of the participants (n = 393).

Age (years)	Frequency	Percent %
40-45	118	30.0
46-50	74	18.8
51-55	53	13.5
56-60	78	19.8
61-65	70	17.8
Gender		
male	131	33.3
Female	262	66.7
Marital status		
Married	271	69.0
single	45	11.5
divorced	29	7.4
widowed	48	12.2
Working status		
No	199	50.6
Yes	194	49.4
Working hours per week		
Less than 35	120	30.5
35- 40	74	18.8
41-50	29	7.4
51-55	24	6.1
More than 55	25	6.4
Education level		
High school or less	164	41.7
University degree	129	32.8
No formal education	66	16.8
Postgraduate	34	8.7
Smoking		
Non-Smoker	251	63.9
Second hand smoker	56	14.2
Ex smoker	44	11.2
Current smoker	42	10.7
Duration since diagnosis with diabetes (years)		
1-5	158	40.2
6-10	133	33.8
11-15	67	17.0
16-20	35	8.9
Other chronic diseases		
No	212	53.9
Yes	181	46.1
Type of chronic diseases		
Hypertension	125	31.8
Epilepsy	6	1.5
Asthma	33	8.4
Anaemia	28	7.1
Hyperthyroidism	10	2.5
Hypothyroidism	47	12.0
Complications		
Diabetic foot	35	8.9
Retinopathy	80	20.4
Numbness in arms and feet	137	34.9
Chronic kidney disease	11	2.8
Stroke	12	3.1
Heart attack	28	7.1
Other medications		
No	218	55.5
Yes	175	44.5
Frequency of diabetes medications per day		
1	71	18.1
2-4	306	77.9
5	16	4.1
Number of chronic disease medications per day		
0	218	55.5
1-3	135	34.4
4-6	32	8.1
7-9	8	2

**Table 2** shows the level of adherence among the participants. Overall, adherence was moderate to high for several behaviours: most participants carried their medications during travel (88.9%) and refrained from self-adjusting doses without consultation. However, a substantial proportion reported non-adherent behaviours, including forgetting doses at least rarely (52.4%), self-modifying prescribed amounts (32.6%), and reducing doses during sick days (22.1%). Financial constraints (21.9%) and side effects (20.9%) were also reported as barriers to full adherence. As reported in **Table 1**, most participants were prescribed 2–4 diabetes medications per day (77.9%), and the most frequently reported complications were peripheral neuropathy (34.9%) and retinopathy (20.4%).

**Table 2:** Responses to the direct adherence questions using the IADMAS.

Item	Always	Often	Sometimes	Rarely	Never
1. During the last month, how many times did you forget to take your medication(s)?	22 (5.6%)	45 (11.5%)	76 (19,3%)	63 (16%)	187 (47.6%)
2. During the last month, how often did you take your medications deliberately in a different dose than what was prescribed for you?	4 (1%)	22 (5.6%)	62 (15.8%)	40 (10.2%)	265 (67.4%)
3. During the last month, how often did you take your medications deliberately at a different time than was prescribed for you?	18 (4.6%)	37 (9.4%)	90 (22.9%)	50 (12.7%)	198 (50.4%)
Item	Yes		No		
4. During the last month, did you take your medication(s) with you when leaving the house (e.g., when visiting relatives or travelling)?	348 (88.9%)		45 (11.5%)		
5. During the last month, did you stop taking your medication(s) without seeking medical consultation because of side effects?	82 (20.9%)		311 (79.1%)		
6. During the last month, did you take a smaller amount of your medication(s) without seeking medical consultation because you felt better	82 (20.9%)		311 (79.1%)		
7. During sick days (such as influenza or diarrhoea), did you take a smaller amount of your medication(s) without seeking medical consultation because of decreased appetite?	87 (22.1%)		306 (77.9%)		
8. During the last month, did you take your medication(s) in lesser amounts because it was expensive?	86 (21.9%)		307 (78.1%)		

IADMAS, Iraq Anti-Diabetic Medication Adherence Scale.

**Table 3** presents Chi-square analyses of factors associated with suboptimal adherence. Five factors were statistically significant (all p < 0.05): absence of treatment plan clarification (p = 0.02), inadequate explanation of diabetes and its complications (p = 0.01), lack of commitment to medication schedules (p = 0.01), discontinuation of medication without medical advice (p < 0.001), and self-reduction of doses during fasting without consultation (p < 0.001). Among those with no commitment to either medication dispensing dates or doctor appointments, 96.8% had suboptimal adherence, compared with 80.6% committed only to dispensing dates and 73.6% committed only to doctor appointments. Demographic factors including age, gender, marital status, and smoking status were not significantly associated with adherence. Full data are presented in **Table 3**.

**Table 3:** Demographic and socioeconomic factors associated with medication adherence among participants.

Category	Suboptimal adherence n (%)	Optimal adherence n (%)	Value of chi-square (p-value)
Age (years)			5.151 (p = 0.27)
40-45	96 (81.4)	22 (18.6)	
46-50	61 (82.4)	13 (17.6)	
51-55	37 (69.8)	16 (30.2)	
56-60	63 (80.8)	15 (19.2)	
61-65	51 (72.9)	19 (27.1)	
Gender			2.710 (p = 0.10)
Male	109 (83.2)	22 (16.8)	
Female	199 (76.9)	63 (24.0)	
Marital status			1.754 (p = 0.63)
Married	210 (77.5)	61 (22.5)	
Single	38 (84.4)	7 (15.6)	
Divorced	24 (82.8)	5 (17.2)	
Widowed	36 (75.0)	12 (25.0)	
Are you working?			0.941 (0.33)
Yes	156(80.4)	38(19.6)	
No	152(76.4)	47(23.6)	
How many hours do you work per week?			9.320 (0.09)
Less than 35h	102(85.0)	18 (15)	
35h-40h	61(82.4)	13(17.6)	
41h-50h	23 (79.3)	6 (20.7)	
51h-55h	15 (62.5)	9 (37.5)	
More than 55h	17 (68.0)	8 (32.0)	
How much do you earn monthly?			3.944 (p = 0.27)
Less than 5000	149(78.8)	40(21.2)	
5000-10000	71(83.5)	14(16.5)	
10001-15000	52(77.6)	15(22.4)	
More than 20000	36(69.2)	16(30.8)	
Education Level			5.718 (0.13)
High school or less	133 (81.1)	31 (18.9)	
Undergraduate	105 (81.4)	24 (18.6)	
No formal education	47 (71.2)	19 (28.8)	
Postgraduate	23 (67.6)	11 (32.4)	
Are you smoking?			7.321 (p = 0.06)
Non-Smoker	197 (78.5)	54 (21.5)	
In contact with smoker	38 (67.9)	18 (32.1)	
Previous smoker	35 (79.5)	9 (20.5)	
Current smoker	38 (90.5)	4 (9.5)	
Do you suffer from any other chronic?			1.423 (0.23)
No	171 (80.7)	41 (19.3)	
Yes	137 (75.7)	44 (24.3)	
Do you take medication other than diabetes medication?			1.047 (0.31)
No	175 (80.3)	43 (19.7)	
Yes	133(76.0)	42(24.0)	
If yes, how many?			2.443 (0.49)
0	175 (80.3)	43 (19.6)	
1-3	102 (75.6)	33(24.4)	
4-6	26 (81.2)	6 (18.8)	
7-9	5 (62.5)	3(37.5)	
How long have you been diagnosed with diabetes?			1.183 (0.76)
1-5	121 (76.6)	37 (23.4)	
6-10	108 (81.2)	25 (18.8)	
11-15	51 (76.1)	16 (23.9)	
16-20	28 (80.0)	7 (20.0)	
How many times do you take diabetes medications per day?			2.207 (0.33)
1	51 (71.8)	20(28.2)	
2-4	244(79.7)	62(20.3)	
5	13(81.2)	3(18.8)	
Do you have reminder?			1.375 (0.24)
No	223(79.9)	56(20.1)	
Yes	85(74.6)	29(25.4)	
If the answer is, yes? mention it			2.886 (0.41)
Alarm	50(74.6)	17(25.4)	
Daily pill Organizer	4(50.0)	4(50.0)	
Family member	20(280.0)	5(20.0)	
Meals	6(75.0)	2(25.0)	
Who provides emotional support for DM?			7.053 (0.13)
Family	203(76.9)	61(23.1)	
Friends	14(73.7)	5(26.3)	
Doctor	22(68.7)	10(31.2)	
Don’t Receive	63(88.7)	8(11.3)	
Other	6(85.7)	1(14.3)	
Which of the following are you committed to?			9.321 (p = 0.01*)
Medication dispensing dates	133(80.6)	32(19.4)	
Doctor appointments	145(73.6)	52(26.4)	
No commitment	30(96.8)	1(3.2)	
Was your treatment plan discussed and clarified by the medical team?			5.141 (p = 0.02*)
No	42(91.3)	4(8.7)	
Yes	266(76.7)	81(23.3)	
Have diabetes and its complications explained by medical team?			7.062 (p = 0.01*)
No	59(90.8)	6(9.2)	
Yes	249(75.9)	79(24.1)	
Have you stopped any medications without medical advice because they were not useful?			9.836 (p < 0.001*)
No	262(75.9)	83(24.1)	
Yes	46(95.8)	2(4.2)	
During the fast day, did you take medications in less than their quantity without medical advice?			8.136 (p < 0.001*)
No	200(74.3)	69(25.7)	
Yes	108(87.1)	16(12.9)	

*= P<=0.05 statistically significant value affecting diabetes treatment adherence.

**Table 4** shows the results of univariate binary logistic regression analysis. Two variables were associated with increased odds of suboptimal adherence: absence of treatment plan clarification by the medical team (crude OR 3.197; 95% CI 1.113–9.186; p = 0.03) and failure to receive explanations about diabetes and its complications (crude OR 3.120; 95% CI 1.298–7.500; p = 0.01). Four variables were associated with reduced odds of suboptimal adherence: not reducing medication doses during fasting without medical advice (crude OR 0.429; 95% CI 0.238–0.776; p < 0.001), commitment to medication dispensing dates (crude OR 0.139; 95% CI 0.018–1.054; p = 0.05), adherence to scheduled doctor appointments (crude OR 0.093; 95% CI 0.012–0.699; p = 0.02), and not discontinuing medication without medical advice (crude OR 0.13; 95% CI 0.033–0.578; p < 0.01). These are unadjusted estimates and should not be interpreted as independent predictors.

**Table 4:** The crude odds ratios (ORs) and 95% confidence intervals from univariate logistic regression analysis of factors associated with suboptimal medication adherence (ORs >1 indicate increased odds of suboptimal adherence; ORs <1 indicate a protective association).

Variable	OR	95% CI	P-value
Which of the following are you committed to?			
- Medication dispensing dates	0.139	0.018-1.054	0.05
- Doctor appointments	0.093	0.012-0.699	0.02
- No commitment	Reference		
Was your treatment plan discussed and clarified by the medical team before it was approved?			
- No	3.197	1.113-9.186	0.03
- Yes			
Have diabetes and its complications been explained to you by the medical team?			
No	3.120	1.298-7.500	0.01
Yes			
During the past month, have you stopped any of your medications without medical advice because you felt they were not useful?			
No	0.13	0.033-0.578	0.00
Yes			
During the fast day, did you take medications in less than their quantity without medical advice?			
No	0.429	0.238-0.776	0.00
Yes			

OR, odds ratio; CI, confidence interval.

**Table 5** presents the results of multivariate backward stepwise binary logistic regression, identifying independent predictors of suboptimal adherence after adjustment for all variables significant in univariate analysis. Four variables retained independent significance in the final model. Among age groups, patients aged 51–55 years had significantly lower adjusted odds of suboptimal adherence compared with patients aged 40–45 years (aOR 0.235; 95% CI 0.065–0.845; p = 0.027). Second-hand smoke exposure was independently associated with reduced adjusted odds of suboptimal adherence compared with non-smokers (aOR 0.295; 95% CI 0.100–0.873; p = 0.027). Working more than 55 hours per week was independently associated with reduced adjusted odds of suboptimal adherence compared with working less than 35 hours per week (aOR 0.141; 95% CI 0.022–0.900; p = 0.038). A diabetes diagnosis duration of 6–10 years was independently associated with increased adjusted odds of suboptimal adherence compared with a duration of 1–5 years (aOR 2.553; p = 0.047). All other variables, including absence of treatment plan clarification, lack of explanation of diabetes complications, commitment to appointments and medication dispensing dates, and unsanctioned medication discontinuation, did not retain independent significance after mutual adjustment.

**Table 5:** Adjusted odds ratios (aORs) and 95% confidence intervals from multivariate backward stepwise binary logistic regression analysis of independent predictors of suboptimal medication adherence (aORs >1 indicate increased odds of suboptimal adherence; aORs <1 indicate a protective association).

Variable	Estimate	SE	Z	aOR	95% CI	P-value
Intercept	2.7033	2.320	1.1651	14.929	0.158-1409.092	0.244
Age (years)						
46-50	-0.4609	0.555	-0.8310	0.631	0.213-1.871	0.406
51-55	-1.4491	0.653	-2.2178	0.235	0.065-0.845	0.027*
56-60	-0.4359	0.639	-0.6823	0.647	0.185-2.262	0.495
61-65	-0.2923	0.796	-0.3670	0.747	0.157-3.556	0.714
40-45	Reference					
Gender						
Female	0.5230	0.517	1.0108	1.687	0.612-4.652	0.312
Male	Reference					
Maital status						
Single	0.2443	0.665	0.3673	1.277	0.347-4.700	0.713
Divorced	1.7623	0.970	1.8164	5.826	0.870-39.011	0.069
Widowed	-1.4173	0.744	-1.9055	0.242	0.056-1.041	0.057
Married	Reference					
Are you working?						
Yes	0.5028	0.574	0.8765	1.653	0.537-5.090	0.381
No	Reference					
How many hours do you work per week?						
35h-40h	-0.1113	0.636	-0.1751	0.895	0.257-3.110	0.861
41h-50h	-0.1628	0.760	-0.2143	0.850	0.192-3.766	0.830
51h-55h	-1.5622	0.932	-1.6761	0.210	0.034-1.303	0.094
More than 55h	-1.9579	0.945	-2.0709	0.141	0.022-0.900	0.038*
Less than 35h	Reference					
How much do you earn monthly?						
5000 - 10000	-0.2059	0.604	-0.3406	0.814	0.249-2.662	0.733
10001-15000	-0.4408	0.671	-0.6568	0.644	0.173-2.398	0.511
More than 20000	0.2331	0.814	0.2862	1.262	0.256-6.227	0.775
Less than 5000	Reference					
Education Level						
High school or less	-0.3525	0.745	-0.4731	0.703	0.163-3.028	0.636
Undergraduate	-0.3655	0.789	-0.4633	0.694	0.148-3.257	0.643
Postgraduate	-1.0394	0.948	-1.0964	0.354	0.055-2.268	0.273
No formal education	Reference					
Are you smoking?						
In contact with smoker	-1.2194	0.553	-2.2058	0.295	0.100-0.873	0.027*
Previous smoker	-0.0432	0.685	-0.0631	0.958	0.250-3.667	0.950
Current smoker	0.8835	0.809	1.0918	2.419	0.495-11.818	0.275
Non-Smoker	Reference					
Do you suffer from any other chronic?						
No	0.1236	0.538	0.2298	1.132	0.394-3.247	0.818
Yes	Reference					
Do you take medication other than diabetes medication?						
No	1.7085	1.531	1.1161	5.521	0.275-110.902	0.264
Yes	Reference					
If yes, how many?						
1-3	1.7219	1.488	1.1575	5.595	0.303-103.300	0.247
4-6	1.9234	1.702	1.1302	6.844	0.244-192.271	0.258
7-9	.	.	.	.	.	.
0	Reference					
How long have you been diagnosed with diabetes?						
6-10	0.9374	0.471	1.9894	2.553	NaN	0.047*
11-15	0.3099	0.670	0.4626	1.363	1.014-6.429	0.644
16-20	0.6727	0.926	0.7266	1.960	0.367-5.069	0.467
1-5						
How many times do you take diabetes medication per day?						
2-4	0.0437	0.527	0.0831	1.045	0.319-12.029	0.934
5	0.9108	1.454	0.6266	2.486	0.372-2.933	0.531
1	Reference					
Do you have reminder?						
No	0.2229	0.428	0.5210	1.250	0.144-42.933	0.602
Yes	Reference					
Who provides emotional support for DM?						
Your friends	0.7182	0.780	0.9211	2.051	0.540-2.891	0.357
Your doctor	0.0512	0.673	0.0760	1.052	0.445-9.453	0.939
You don’t receive any support	0.8528	0.646	1.3202	2.346	0.281-3.937	0.187
Others	1.1596	1.352	0.8580	3.189	0.662-8.321	0.391
Your Family	Reference					
Which of the following are you committed to?						
Doctor appointments	-2.0513	1.268	-1.6177	0.129	0.226-45.082	0.106
Medication dispensing dates	-1.4010	1.281	-1.0934	0.246	0.011-1.543	0.274
No commitment	Reference					
Was your treatment plan discussed and clarified by the medical team?						
No	0.2581	1.100	0.2347	1.294	0.020-3.036	0.814
Yes	Reference					
Have diabetes and its complications explained by medical team?						
No	1.4543	1.049	1.3861	4.281	0.150-11.173	0.166
Yes	Reference					
Have you stopped any medication without medical advice because they were not useful?						
No	-1.7925	1.242	-1.4434	0.167	0.548-33.471	0.149
Yes	Reference					
During the fast day, did you take medications in less than their quantity without medical advice?						
No	0.2411	0.523	0.4613	1.273	0.015-1.899	0.645
Yes	Reference					

OR, odds ratio; CI, confidence interval.

*= P<=0.05 statistically significant value affecting diabetes treatment adherence.

## Discussion

Medication adherence is vital for managing type 2 diabetes, as poor adherence leads to complications and higher mortality (14). This study examined factors affecting adherence among patients at King Abdulaziz University Hospital, KSA, in line with previous research. Non-adherence in T2DM is a multifactorial phenomenon shaped by patient-related factors (health beliefs, perceived medication necessity, and illness burden), therapy-related factors (regimen complexity, polypharmacy, and side effects), and healthcare system factors (quality of patient–provider communication and continuity of follow-up). The World Health Organization has identified non-adherence as one of the foremost challenges in chronic disease management globally, noting that improvements in adherence produce larger health gains than many advances in specific pharmacological treatments. Globally, adherence rates in T2DM rarely exceed 50%, with suboptimal adherence documented across high-, middle-, and low-income countries, including the Gulf region, South Asia, sub-Saharan Africa, and Europe.

The study revealed that 78.4% of participants had suboptimal adherence, whereas only 21.6% demonstrated optimal adherence. This finding is consistent with a previous study that reported 74.9% suboptimal and 25.1% optimal adherence (13). This suggests that our study exhibited a slightly higher rate of suboptimal adherence and a lower rate of optimal adherence. Such differences may be attributed to the characteristics of our study population, as all participants were aged 40 years or older, a demographic associated with lower adherence owing to cumulative health complications and forgetfulness. Furthermore, a higher proportion of female participants and individuals with no formal education in our study, compared to other study (13), could have contributed to the observed lower adherence rates. Therefore, these findings emphasise the importance of addressing educational gaps and tailoring interventions to specific demographic and socio-cultural contexts to improve adherence. The higher rate of suboptimal adherence observed in this sample compared with prior Saudi studies may also reflect the complexity of managing multiple chronic conditions simultaneously. Polypharmacy — a common feature among middle-aged T2DM patients with comorbidities — is consistently associated with reduced adherence in international literature, with each additional daily medication increasing the cognitive and logistical burden on the patient. Studies from Tanzania, Japan, and Spain similarly confirm that regimen complexity is a major structural barrier to adherence, independent of patient motivation or disease knowledge, underscoring the need for simplified regimens and enhanced pharmacy counselling. Multivariate backward stepwise logistic regression identified four independent predictors of suboptimal adherence after adjustment for all covariates. Patients aged 51–55 years had significantly lower adjusted odds of suboptimal adherence than those aged 40–45 years (aOR 0.235; 95% CI 0.065–0.845; p = 0.027), suggesting that adherence may improve through middle age before other age-related barriers emerge. Exposure to second-hand smoke was independently associated with reduced adjusted odds of suboptimal adherence compared with non-smokers (aOR 0.295; 95% CI 0.100–0.873; p = 0.027), a counterintuitive finding that may reflect health-monitoring behaviours among patients aware of their combined cardiovascular risk. A diabetes diagnosis duration of 6–10 years was independently associated with increased adjusted odds of suboptimal adherence compared with a duration of 1–5 years (aOR 2.553; p = 0.047), consistent with the hypothesis that patients may experience treatment fatigue or reduced perceived benefit after the initial period of disease management. Working more than 55 hours per week was independently associated with reduced odds of suboptimal adherence compared with working fewer than 35 hours (aOR 0.141; 95% CI 0.022–0.900; p = 0.038), possibly reflecting greater self-discipline or access to workplace health support among higher-intensity workers. Notably, several factors that emerged as significant in univariate analysis — including absence of treatment plan clarification, lack of explanation of diabetes complications, and unsanctioned medication discontinuation — did not retain independent significance after mutual adjustment, indicating that their apparent effects may be mediated or confounded by the sociodemographic variables that remained significant in the multivariate model.

Regarding gender, studies conducted in Jeddah revealed a statistically significant association between male gender and higher adherence levels (13). Conversely, in the current study and a study from the Eastern Province of Saudi Arabia, females reported higher adherence levels compared to males (4). Although the p-value for gender in this study indicates no statistical significance, the study from the Eastern Province found a significant association, highlighting an intriguing discrepancy in the findings (4).

A study conducted in the Eastern Province of Saudi Arabia revealed that nearly half of the participants identified a lack of understanding about diabetes and the importance of its management as the second most significant factor influencing medication adherence (15). Similarly, the current study found that patients who did not receive adequate explanations about diabetes, its complications, and the treatment plan from the medical team exhibited suboptimal adherence. The mechanistic link between health literacy and adherence is well established: patients who understand their disease, the rationale for treatment, and the consequences of non-adherence are more likely to internalise medication-taking as a necessary self-management behaviour. The Health Belief Model posits that perceived susceptibility to complications and perceived benefit of treatment are among the strongest determinants of adherence behaviour, directly supporting the present finding. Structured diabetes self-management education (DSME) programmes have demonstrated efficacy in improving adherence and glycaemic control in randomised controlled trials conducted in the United States, the United Kingdom, and across the Middle East, and their integration into routine outpatient care in Saudi tertiary hospitals warrants prioritisation. In contrast, a study in the Asir region demonstrated that patients with good awareness of type 2 diabetes exhibited optimal adherence (12). Therefore, enhancing knowledge about diabetes is crucial and can be achieved through various methods, such as utilizing social media platforms for education, encouraging participation in diabetes support groups, and organizing targeted educational campaigns to raise awareness (15).

A study on a Muslim population reported that severe hypoglycaemia occurred more frequently during the fasting period of Ramadan, primarily due to changes in treatment regimens during that time (14). Similarly, the current study highlights that patients often reduced their medication doses during fasting days without seeking medical advice, contributing to poor compliance with their prescribed regimens. Hence, managing changes in medication schedules during Ramadan by healthcare providers to adjust treatment plans is essential to ensure safe fasting and adherence to medication. Internationally, Ramadan-specific diabetes management guidelines have been developed by major diabetes societies, including the International Diabetes Federation (IDF) and the American Diabetes Association (ADA), which recommend pre-Ramadan risk assessment, structured education, and individualised dose adjustment. The mechanistic pathway through which Ramadan fasting impairs adherence includes disrupted circadian rhythm of meals and medications, altered insulin sensitivity, and psychological reluctance to take medications that are perceived as invalidating the fast. These factors highlight the need for proactive, culturally sensitive healthcare consultations ahead of the fasting period.

The quality of the treatment relationship has been recognized as a key factor influencing adherence (16). Previous studies have shown that a lack of commitment to medication refills and follow-up appointments with doctors is significantly associated with suboptimal adherence levels (13). Likewise, in the current study, a significant relationship was found between lack of commitment to follow-up appointments and medication refills and suboptimal adherence levels. In light of these results, it may be beneficial to provide personalised follow-up support, such as dedicated calls or messages to remind patients of their appointments and medication refills, while offering flexible scheduling options to accommodate their needs. Such support could help patients feel more engaged and improve their long-term adherence to treatment. The mechanistic role of the patient–provider relationship in adherence operates through several pathways: shared decision-making enhances patients’ sense of ownership over their treatment plan; continuity of care with the same provider fosters trust and open communication about side effects and concerns; and structured follow-up provides regular reinforcement of adherence behaviours. Evidence from the United Kingdom, Canada, and Australia consistently demonstrates that patients receiving coordinated care through nurse-led diabetes clinics or structured diabetes management programmes show significantly higher adherence rates than those receiving standard outpatient consultations, suggesting that systemic changes in care delivery may be as important as individual patient-level interventions.

We also found a statistically significant association between unsanctioned medication discontinuation and the belief that the medication was ineffective. This finding implies that adherence behaviour is substantially influenced by patients’ perceptions of medication efficacy: patients who perceive no therapeutic benefit are more likely to discontinue treatment independently without consulting their healthcare provider. Internationally, similar findings have been reported from Tanzania, Bulgaria, and Japan, where negative medication beliefs consistently ranked among the strongest predictors of non-adherence. This underscores the importance of fostering clear patient–provider communication, addressing misconceptions about medication effectiveness, and building therapeutic trust as core components of diabetes management.

The study conducted across Ministry of Health primary healthcare centres in Jeddah, Saudi Arabia, explored medication adherence among patients with T2DM and noted that smoking was reported among participants but was not significantly associated with adherence (13). Similarly, in the current study, smoking did not emerge as a significant factor, likely owing to the small sample size and the tendency of patients to underreport smoking habits. These findings highlight the importance of employing larger sample sizes and anonymous reporting tools to better assess the impact of smoking on health outcomes.

The current study found no statistically significant association between diabetes duration and medication adherence. In contrast, Rayana et al. reported that longer diabetes duration was associated with higher adherence levels (OR = 3.039, p = 0.0258) (17). Additionally, other studies have shown conflicting results, with some reporting higher adherence among patients with a diabetes duration of ≤5 years (18). It is possible that some patients with a longer duration of diabetes adapt to the condition in ways that reduce their strict reliance on medications, which may decrease adherence compared to those who are newly diagnosed.

This study addressed a critical public health issue by exploring factors influencing treatment compliance among middle-aged patients with type 2 diabetes mellitus (T2DM) in Saudi Arabia, a region with one of the highest global diabetes prevalence rates with a reported prevalence of 28% from previous meta-analysis research (9).Moreover, we used a validated, forward-backward translated questionnaire to ensure reliable data collection and provide a comprehensive analysis of adherence in a tertiary care setting.

This study has several limitations that must be acknowledged. First, the cross-sectional design prevents the establishment of causal relationships between the identified factors and suboptimal adherence; it is not possible to determine whether poor adherence precedes or results from inadequate patient education and follow-up. Longitudinal or interventional studies are required to confirm directionality. Second, the use of a consecutive sampling strategy at a single tertiary-care centre introduces potential selection bias: patients attending King Abdulaziz University Hospital may differ systematically from those managed in primary or community care settings in terms of disease severity, socioeconomic status, and health-seeking behaviour, which limits the generalisability of findings to the broader Saudi T2DM population. Third, adherence was assessed using the IADMAS, a self-report instrument, which is susceptible to social desirability bias — patients may over-report adherent behaviours during face-to-face interviews to appear compliant to the research team. Although the one-month recall window of the IADMAS minimises retrospective distortion, residual recall bias cannot be excluded entirely. Fourth, the study focused exclusively on middle-aged patients (40–65 years) attending outpatient clinics at a single hospital. To address potential selection bias, measures such as including only patients with T2DM who had been receiving care at the diabetes centre for over a year were undertaken. Moreover, data collection was evenly distributed across various physicians’ clinics, likely reducing bias. However, the use of a cross-sectional design restricts causal inferences, and self-reported data may have introduced recall or social desirability biases. Lastly, the classes of antidiabetic medications used by participants were not evaluated in this study.

To mitigate these limitations, future research should adopt multicentre designs spanning both primary and tertiary care, include broader age groups, use objective adherence measures such as pharmacy refill records or electronic monitoring devices alongside self-report tools, and employ longitudinal or mixed-methods designs to elucidate causal mechanisms and patient experiences.

Furthermore, the study sample included a higher proportion of females compared to males, which may limit the generalisability of the findings. Future research should aim to include a more balanced sample or specifically recruit male participants to better understand gender-specific factors influencing adherence.

Medication adherence may be influenced by costs, a factor not evaluated in detail in this study. Although free medications are available at Ministry of Health Primary Health Care Centres (PHCCs), patients at King Abdulaziz University Hospital (KAUH), including residents, may face financial barriers. These constraints, despite free medication programs, remain a key factor affecting adherence and should be explored in future research.

## Conclusion

This study explored factors influencing medication adherence among middle-aged patients with type 2 diabetes at King Abdulaziz University Hospital, finding that 78.4% demonstrated suboptimal adherence. Univariate analysis identified absence of treatment plan clarification, inadequate explanation of diabetes complications, lack of commitment to follow-up appointments and medication dispensing dates, and unsanctioned medication discontinuation as factors associated with suboptimal adherence. After multivariate adjustment, four variables were independently associated with suboptimal adherence: patients aged 51–55 years and those exposed to second-hand smoke had significantly reduced adjusted odds of suboptimal adherence, while a diabetes diagnosis duration of 6–10 years was associated with increased adjusted odds. Working more than 55 hours per week was also independently associated with reduced odds of suboptimal adherence. These findings highlight the complex interplay of sociodemographic and clinical factors that independently determine adherence outcomes and underscore the need for targeted, patient-centred interventions. Further long-term multicentre studies are necessary to confirm these associations and evaluate the sustainability of adherence interventions.

## Data Availability

The raw data supporting the conclusions of this article will be made available by the authors, without undue reservation.

## References

[1] Suzuki R Saita S Nishigaki N Kisanuki K Shimasaki Y Mineyama T . Factors associated with treatment adherence and satisfaction in type 2 diabetes management in Japan: Results from a web-based questionnaire survey. Diabetes Ther. (2021) 12:2343–58. doi: 10.1007/s13300-021-01100-3 34283372 PMC8384993

[2] Gomez-Peralta F Pérez JAF Molinero A Barrancos IMS Martínez EA Martínez-Pérez P . Adherence to antidiabetic treatment and impaired hypoglycemia awareness in type 2 diabetes mellitus assessed in Spanish community pharmacies: The ADHIFAC study. BMJ Open Diabetes Res Care. (2021) 9. doi: 10.1136/bmjdrc-2021-002148 34845061 PMC8633992

[3] Wakui N Ozawa M Yanagiya T Endo S Togawa C Matsuoka R . Factors associated with medication compliance in elderly patients with type 2 diabetes mellitus: A cross-sectional study. Front Public Health. (2022) 9:771593. doi: 10.3389/fpubh.2021.771593 35087782 PMC8787062

[4] Alsaidan AA Alotaibi SF Thirunavukkarasu A ALruwaili BF Alharbi RH Arnous MM . Medication adherence and its associated factors among patients with type 2 diabetes mellitus attending primary health centers of Eastern Province, Saudi Arabia. Med (Lithuania). (2023) 59. doi: 10.3390/medicina59050989 37241220 PMC10223340

[5] Cramer JA Roy A Burrell A Fairchild CJ Fuldeore MJ Ollendorf DA . Medication compliance and persistence: Terminology and definitions. Value Health. (2008) 11:44–7. doi: 10.1111/j.1524-4733.2007.00213.x 18237359

[6] Ekenberg M Qvarnström M Sundström A Martinell M Wettermark B . Socioeconomic factors associated with poor medication adherence in patients with type 2 diabetes. Eur J Clin Pharmacol. (2024) 80:53–63. doi: 10.1007/s00228-023-03571-8 37870618 PMC10781833

[7] Lim D Woo K . Medication adherence and related factors among older adults with type 2 diabetes who use home health care. Geriatr Nurs (Minneap). (2025) 61:270–7. doi: 10.1016/j.gerinurse.2024.11.014 39566237

[8] Doya IF Yahaya JJ Ngaiza AI Bintabara D . Low medication adherence and its associated factors among patients with type 2 diabetes mellitus attending Amana Hospital in Dar es Salaam, Tanzania: a cross-sectional study. Int Health. (2024) 16:200–7. doi: 10.1093/inthealth/ihad042 37310004 PMC10911532

[9] Moradinazar M Babakhani M Rostami R Shakiba M Moradi A Shakiba E . Epidemiological status of type 2 diabetes mellitus in the Middle East and North Africa, 1990–2019. Eastern Mediterr Health J. (2022) 28:478–88. doi: 10.26719/emhj.22.050 35959663

[10] Radi S Alsolami HA Bader MW Almazmumi MK Alsahafi AH Habeeballah JH . Adherence to treatment guidelines among patients with type 2 diabetes attending a tertiary hospital in Jeddah, Saudi Arabia. Cureus. (2024) 16, e73850. doi: 10.7759/cureus.73850 39563693 PMC11576062

[11] Abdelwahab SI Taha MME Kaabi YA . Diabetes mellitus research in Saudi Arabia: A bibliometric study (2010-2021). J Family Med Prim Care. (2023) 12:1038–49. doi: 10.4103/jfmpc.jfmpc_1889_22 37636176 PMC10451595

[12] Alhammadi NA AL Qahtani AA Hosiky MF AL Shahrani FS AL Shehri SM AL Ghamdi MA . Public awareness of diabetes complications and its effect on treatment compliance in Asir region, Saudi Arabia. J Family Med Prim Care. (2022) 11:6812–7. doi: 10.4103/jfmpc.jfmpc_422_22 36992996 PMC10041207

[13] Khayyat YA Alshamrani RM Bintalib DM Alzahrani NA Alqutub S . Adherence to hypoglycemic agents in type 2 diabetes mellitus: A cross-sectional study. Cureus. (2022) 14, e22626. doi: 10.7759/cureus.22626 35371760 PMC8960541

[14] Salti I Bénard E Detournay B Bianchi-Biscay M Le Brigand C Voinet C . A population-based study of diabetes and its characteristics during the fasting month of Ramadan in 13 countries study on behalf of the EPIDIAR study group (2004). Available online at: https://www.aventisLebanon.com (Accessed May 10, 2026). 10.2337/diacare.27.10.2306 15451892

[15] Alfulayw MR Almansour RA Aljamri SK Ghawas AH Alhussain SS Althumairi AA . Factors contributing to noncompliance with diabetic medications and lifestyle modifications in patients with type 2 diabetes mellitus in the Eastern Province of Saudi Arabia: A cross-sectional study. Cureus. (2022) 14:e31965. doi: 10.7759/cureus.31965 36582555 PMC9795535

[16] Tiktin M Celik S Berard L . Understanding adherence to medications in type 2 diabetes care and clinical trials to overcome barriers: A narrative review. Curr Med Res Opin. (2016) 32:277–87. doi: 10.1185/03007995.2015.1119677 26565758

[17] Dinkova R Marinov L Doneva M Kamusheva M . Medication adherence among patients with diabetes mellitus and its related factors—a real-world pilot study in Bulgaria. Med (Lithuania). (2023) 59. doi: 10.3390/medicina59071205 37512017 PMC10383103

[18] Gelaw BK Mohammed A Tegegne GT Defersha AD Fromsa M Tadesse E . Nonadherence and contributing factors among ambulatory patients with antidiabetic medications in Adama Referral Hospital. J Diabetes Res. (2014) 2014. doi: 10.1155/2014/617041 25544946 PMC4269085

